# Neuromodulation of choice-induced preference changes: the tDCS study of cognitive dissonance

**DOI:** 10.3389/fpsyg.2023.1104410

**Published:** 2023-12-18

**Authors:** Elena Rybina, Marco Colosio, Anna Shestakova, Vasily Klucharev

**Affiliations:** Institute of Cognitive Neuroscience, HSE University, Moscow, Russia

**Keywords:** cognitive dissonance, decision making, preference changes, medial frontal cortex (MFC), transcranial direct current stimulation (tDCS), free choice paradigm

## Abstract

**Introduction:**

Difficult choices between two equally attractive options result in a cognitive discrepancy between dissonant cognitions such as preferences and actions often followed by a sense of psychological discomfort known as cognitive dissonance. It can lead to changes in the desirability of options: the chosen option becomes more desirable, whereas the rejected option is devalued. Despite the ample experimental evidence to show this effect, the neural mechanisms and timing of such choice-induced preference changes are not fully understood.

**Methods:**

In this study, we used transcranial direct current stimulation (tDCS) to modulate the activity of the posterior medial frontal cortex (pMFC), which has been associated with conflict monitoring and choice-induced preference changes in neuroimaging studies. Prior to a revised version of Brehm’s free-choice paradigm, participants in two experiments underwent cathodal (inhibitory) or anodal (excitatory) tDCS of the pMFC compared to sham (placebo) stimulation prior to the choice phase.

**Results:**

Our results showed that cathodal tDCS significantly decreased the choice-induced preference change relative to a sham, but only in direct comparisons of rejected options. No significant effect of anodal tDCS in comparison with sham was observed.

**Discussion:**

This study replicates the general behavioral effect of cognitive dissonance and provide partial support for the theory of the pMFC contribution to choice-related cognitive dissonance and subsequent preference changes, with possible limitations of an under-sampling for the obtained effect size and an asymmetry in the inhibitory-excitatory effects of non-invasive tDCS.

## 1 Introduction

Contrary to the assumptions of normative economic theory, choice preferences are not only driven by our attitudes but also modulated by the experience of previous choices. Brehm’s study (1956) suggested that, after choosing between two similarly attractive options, individuals no longer perceive these options as similar, evaluating the chosen option more positively and devaluating the unchosen option. The devaluation of the rejected option has been repeatedly demonstrated in studies using different versions of “free choice paradigm” (Kitayama et al., 2004; Izuma et al., 2010; Mengarelli et al., 2015; Colosio et al., 2017).

According to the prominent theory of cognitive dissonance (Festinger, 1957), “difficult” choices between similarly appealing options that require the rejection of one of them result in a cognitive discrepancy between dissonant cognitions such as preferences and actions. This discrepancy arises from the need to act in a manner that contradicts one’s preferences and attitudes towards the highly favored option and may subsequently lead to a sense of psychological discomfort, also known as dissonance (Harmon-Jones and Mills, 2019). This discomfort motivates individuals to be consistent with their actions and reduce the dissonance by either devaluing the rejected option or increasing their evaluation for selected one. Thus, the mere act of choosing between similarly preferred options affects individual preferences.

In a typical “free choice paradigm,” participants are asked to rate a set of goods according to their preference (*preference task I*). Next, they select between two of the items that had similar preference ratings in the first rating task (*choice task*). Finally, participants are asked to re-rate the original set of goods for the second time (*preference task II*). According to the theory of cognitive dissonance, after making a difficult choice between two equally preferred items, participants’ preference, guided by the need to resolve conflict, can decrease for the rejected item and increase for the chosen ones. The resulting difference between the assessment of the items in *preference task II* and *preference task I* could represent the observable resolution of cognitive dissonance: spreading of alternatives or choice-induced preference changes. Alteration of preference was observed in plenty of studies, either preference devaluation for rejected options (for example, Izuma and Murayama, 2013; Salti et al., 2014; Colosio et al., 2017) or an increase in evaluation for selected ones (for example, Sharot et al., 2010; Izuma and Murayama, 2013).

Importantly, the free-choice paradigm can produce artificial preference changes (see Chen and Risen, 2010; Izuma and Murayama, 2013; Enisman et al., 2021 for a review). Chen and Risen (2010) showed that measured alteration in preference when making a difficult decision may not necessarily be associated with the choice itself; rather, it may be a result of the artifact, while choice and repeated evaluation merely uncover already existing preferences. For example, preference for *option 1*, measured by rating or ranking, can slightly exceed preference for *option 2*, although the ratings were equal during *preference task I*. Therefore, it is likely that in *preference task II*, preference rating for *option 1* will continue to get even higher, producing ostensible changes of preference. To counter this drawback of the “free-choice paradigm,” various control conditions and task modifications have been suggested and investigated (Chen and Risen, 2010; Izuma and Murayama, 2013; Enisman et al., 2021). The use of the brain stimulation approach also may overcome the limitations of the free-choice paradigm. Alterations in preference in making difficult conflictual decisions under region-specific brain stimulation may be attributed to the suppression or enhancement of the neuronal activity in the region responsible for conflict monitoring and resolution, and cannot be attributed to a statistical artifact. Therefore, the substantial effect of well-controlled brain stimulation on the following conflictual decision spreading of alternatives is likely attributable solely to the modulation of neural mechanisms underlying choice-induced preference changes.

Despite the significant progress in studying cognitive dissonance, neurocognitive mechanisms of preference alteration in decision making are still not fully understood. Several studies consistently indicated the involvement of the pMFC (van Veen et al., 2009; Izuma et al., 2010), posterior cingulate cortex (Kitayama et al., 2013; Tompson et al., 2016), dorsolateral prefrontal cortex (Harmon-Jones et al., 2008; Flavia Mengarelli et al., 2015), and nucleus accumbens (Izuma et al., 2010; Kitayama et al., 2013) to post-decisional preference changes. The involvement of other brain regions was not replicated. It is likely these brain regions form a network responsible for detection dissonance and its subsequent resolution (Colosio et al., 2018; Voigt, 2022).

A growing number of studies indicate the critical role of the posterior medial frontal cortex (pMFC) in cognitive dissonance and preference re-evaluation (van Veen et al., 2009; Izuma et al., 2010; Colosio et al., 2017; Voigt et al., 2019; Tandetnik et al., 2021). This part of the brain largely consists of the pre-supplementary motor area (pre-SMA), the dorsal medial frontal cortex (dmPFC), ventral medial frontal cortex (Voigt et al., 2019), and the dorsal anterior cingulate cortex (dACC) (Izuma, 2013; Tandetnik et al., 2021). A number of fMRI studies consistently showed activations in Brodmann areas 10/24/32 in both left and right hemispheres (Izuma, 2013). The pMFC has been associated with monitoring of conflicts, cognitive control, error detection (Carter et al., 1998; Botvinick et al., 1999, 2001; Holroyd Nieuwenhuis et al., 2003; Danielmeier et al., 2011), and reward-based decision making (Williams et al., 2004). Overall, pMFC activity has been linked to performance monitoring and behavior adjustment. Recently, neuroimaging studies have focused on the role of the pMFC in cognitive dissonance and following the difficult choice preference changes (van Veen et al., 2009; Izuma et al., 2010; Jarcho et al., 2011; Kitayama et al., 2013; Voigt et al., 2019). A multichannel electroencephalographic (EEG) study demonstrated that the fronto-central resting state activity predicted the individual strength of preference changes and the magnitude of the dissonance-related neural activity (Colosio et al., 2017). The newest fMRI study showed partitioning of the activity of the medial frontal cortex: vmPFC is associated with expected reward-based decision making, whereas dmPFC is linked with metacognitive aspects of decisions such as deliberation and confidence about the alternatives and choice (Clairis and Pessiglione, 2022). Thus, the activity of the medial frontal cortices at rest affects different aspects of the behavioral effects of cognitive dissonance.

The use of a neuromodulatory approach with the help of repetitive transcranial magnetic stimulation (rTMS) (Izuma et al., 2015) facilitated the unveiling of the causal role of the pMFC in generating and reducing cognitive dissonance in a modified “free-choice paradigm” with a “choice-blindness” procedure. A disruption of pMFC activity, using 1 Hz rTMS right after the choice stage of the free-choice paradigm, significantly reduced the choice-induced preference changes. Although the rTMS approach demonstrated great potential in elucidating the causal relationship between cortical areas, the temporal aspect of the rTMS precludes one from understanding whether choice-induced preference changes take place during *preference task II* or the *choice task*. In the past decade, functional neuroimaging studies (e.g., Izuma et al., 2010) have explored the neural underpinning of cognitive dissonance, predominantly during the post-decisional stage of the “free choice paradigm” when subjects rated options again, some time after making difficult choices (Izuma and Murayama, 2013). This is based on the theoretical proposition that cognitive dissonance is experienced after making a difficult decision, which subsequently leads to an increase in preference for the chosen highly attractive option and a decrease in preference for the rejected one. Importantly, the activity of the pMFC was demonstrated already during the making of such a decision (*choice task*) (Kitayama et al., 2013; Voigt et al., 2019), which supports the hypothesis about the occurrence of preference changes while making a choice. An EEG study with use of “free choice paradigm” demonstrated that difficult decisions during the choice task are associated with stronger evoked elevated activity in the pMFC, reflected in a larger fronto-central error-related negativity (ERN) response, compared to easy decisions (Colosio et al., 2017). A comparison of ERN amplitude between trials featuring difficult and easy choices revealed that the ERN amplitude was higher for difficult ones. Furthermore, the ERN amplitude correlated with the magnitude of choice-induced preference changes. The difference waves (trials in difficult choices versus trials in easy choices) in Cz electrodes position significantly correlated with the extent of spread of alternatives. Thus, a stronger ERN was observed in the Choice task, and the stronger individual preferences were later altered for rejected items in Preference task II (Colosio et al., 2017). Since ERN activity was manifested during choices, the above-mentioned results suggest that the pMFC may be involved in the preference changes at an earlier stage than previously thought. This hypothesis about alteration of preference at an early stage is also supported by the studies of metacognitive aspects of choices (Lee and Daunizeau, 2020; Lee and Holyoak, 2021; Clairis and Pessiglione, 2022).

In this study, we applied transcranial direct current stimulation (tDCS) over the pMFC to probe the critical role of the pMFC in choice-induced preference changes and its contribution to cognitive dissonance during decision-making. The tDCS is a non-invasive neuromodulation technique that temporarily enhances (more often, anodal stimulation) or reduces (more often, cathodal stimulation) cortical excitability. This effect is achieved through applying a constant weak electrical current through an electrode placed on the surface of the scalp. Importantly, tDCS may result in facilitation of, or interference with the targeted brain region activity underlying changes of behavior (Nitsche and Paulus, 2001; Nitsche et al., 2008; Brunoni et al., 2012). This technique has been recently employed to explore the role of the medial frontal cortex in the modulation of error processing and performance monitoring (i.e., the modulation of the ERN and feedback-related negativity) in both clinical (Reinhart et al., 2015) and healthy populations (Bellaïche et al., 2013; Reinhart and Woodman, 2014). Here, we have conducted two sham-controlled experiments with delivering cathodal tDCS of the pMFC (Experiment 1) and anodal tDCS of the pMFC (Experiment 2). Using tDCS, we do not anticipate any effect of brain stimulation on ostensible preference changes due to statistical artifact found by Chen and Risen (2010) for *option 1* and *option 2*. Therefore, any significant differences in preference changes across stimulation conditions could be predominantly attributed to the causal role of the pMFC in evoked by difficult choices spread of alternatives.

By applying tDCS at the preliminary decision stage of the “free choice paradigm,” we expected to exert control on the cortical excitability of the pMFC, and thus observe either a reduction (after cathodal stimulation) or an increase (after anodal stimulation) of the choice-induced preference changes compared to the non-stimulated (sham tDCS) condition, particularly after making difficult choices. The previous study by Colosio et al. (2017) demonstrated more explicit and accurately interpreted alteration of preference after hard choices specifically for the options which were rejected. Therefore, to specify hypothesis and test the stimulation effect, in this study, we were mainly interested in the alteration of preference for rejected option under tDCS, expecting a decrease in the devaluation of the attitude towards declined options in difficult decisions.

## 2 Materials and methods

### 2.1 Participants

Two groups of healthy right-handed volunteers were invited to participate in one of two experiments. Taking into consideration a little knowledge about neuromodulatory effects on the activity of the pMFC using the non-invasive tDCS in the cognitive dissonance theory, we took an averaged group number (17–20 participants) based on the studies with similar design. For the experiment with cathodal tDCS stimulation (Experiment 1), we recruited 18 volunteers. One of them was excluded due to a distraction during the experiment, leaving 17 participants in total (mean age = 22.15, 9 males). For the experiment with anodal tDCS (Experiment 2), we recorded the data of 24 participants. We excluded five participants from the analysis due to the following reasons: (1) one participant had a technical problem with the software; (2) another participant experienced highly uncomfortable sensations from tDCS; (3) three participants reported strong fatigue. Thus, for Experiment 2, we analyzed the results of 19 participants (mean age = 23 years, 9 males).

All participants were instructed to fast at least 3 h before each session. All participants were naïve to tDCS and the nature of the experiment; they were not informed about the protocol received (i.e., sham or stimulation). Participants were recruited through posted advertisements and participated in this experiment in exchange for a small monetary compensation (equivalent to ~10 USD). All volunteers had a normal or corrected-to-normal vision and took no regular medications. None of the subjects had a history of neurological or psychiatric illness. The study protocol was approved by the Institutional Review Board of the HSE of the National Research University Higher School of Economics (Statement of Opinion on compliance of the Empirical Research Project with ethical norm). All participants gave informed written consent before entering the study.

### 2.2 Transcranial direct current stimulation procedure

Each participant received both an active and sham stimulation in two different experimental sessions. Within each group, participants were randomly assigned to receive either tDCS (cathodal tDCS in Experiment 1 or anodal tDCS in Experiment 2) or control (sham) stimulation during the first session, whereas the remaining stimulation was delivered during the second session a week later. The tDCS protocols were based on the safety guidelines (Antal et al., 2017).

The tDCS was applied using a battery-driven 8-channel constant current neuro-stimulator (Startstim 8, Neuroelectrics) and two conductive rubber electrodes hosted in saline-soaked synthetic sponges (active electrode, 19.25 cm^2^; reference, 52 cm^2^). The active electrode was placed over the medial-frontal cortex (FCz position of the international EEG 10–20 system) and held in place by a neoprene headcap, while the reference electrode was placed diagonally at the center of the right cheek.

For active stimulation, the current was increased over the first 30 s. Then cathodal or anodal direct current was delivered constantly for 20 min at an intensity of 1.5 mA. This protocol has been successfully used to down-regulate the medial frontal cortex and associated ERN component (see Reinhart and Woodman, 2014, for details of the current flow model). The impedance was controlled by *Neuroelectrics Instrument Controller* software v1.4, (NIC, Neuroelectrics) and was kept below 10 kΩ. After 20 min of stimulation, the current was ramped down over 30 s. The sham tDCS stimulation was administered following the same procedure as the active tDCS stimulation, but stimulation lasted only 30 s, ramping up and down at the beginning and the end of the 20 min period, producing the same tingling sensations associated with active stimulation. Such a sham stimulation protocol has been shown to be a reliable control condition in both naïve and experienced participants (Gandiga et al., 2006).

### 2.3 Stimuli

Two sets of 223 digital (sets A or B), colorful photos of snack foods on a white background (chocolate, chips, small fruit or vegetable, cheese, etc.) were used as stimuli. We counterbalanced sets A and B across stimulation conditions. To ensure that both sets of stimuli contained similarly attractive items, we used ratings provided by 45 participants (20 males, mean age of 22.17) during our previous experiment (see Colosio et al., 2017, for details) to determine the average preference of each item. Then we assigned items to set A or B in such a way that both sets would consist of the same number of items, and item ratings would show similar distributions and standard deviations (see the results section for statistics).

The photos were projected onto a screen with a visual angle of 4.772^o^ vertically and 7.62^o^ horizontally.

### 2.4 Experimental design

**Figure 1:**
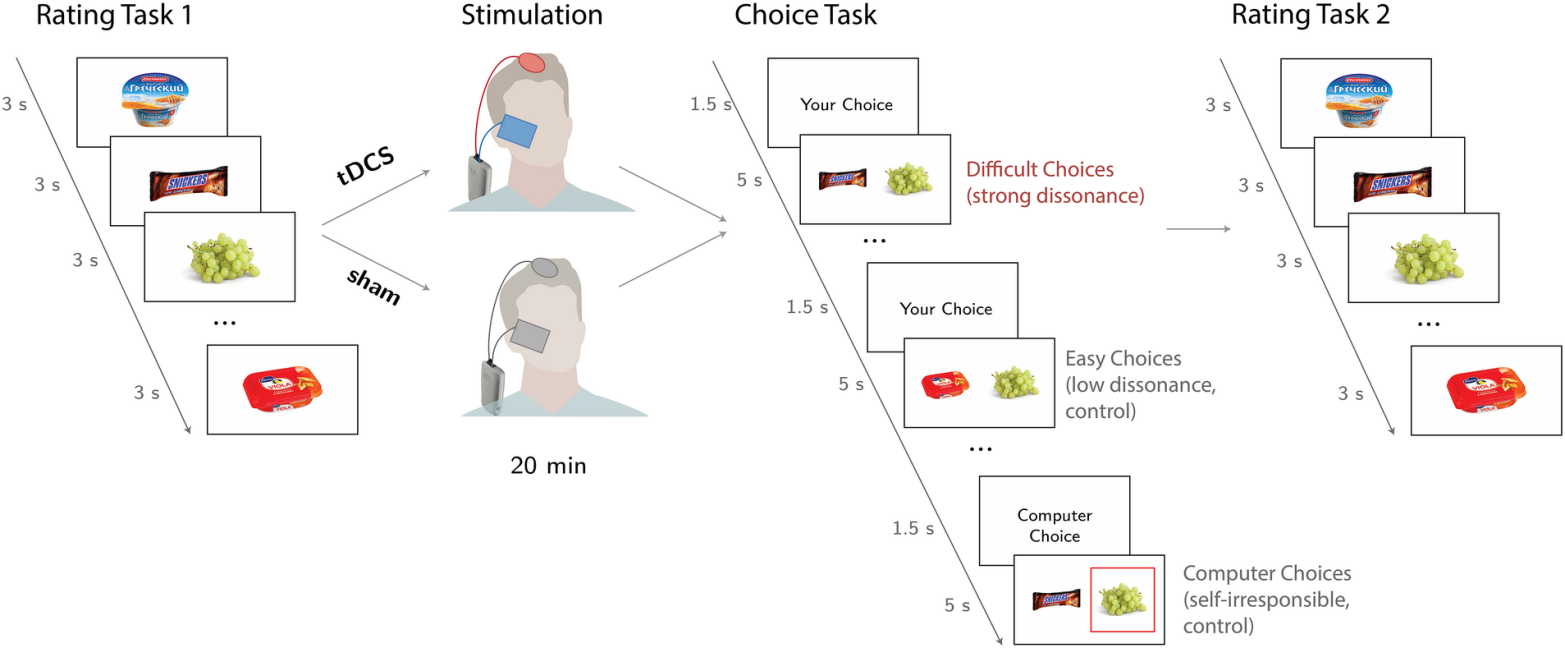
“Free-choice paradigm” used in the study. During preference task I (Rating task 1), participants rated food items presented on the screen for three seconds. Next, during the Choice Task, subjects freely selected one of two food items (self-difficult trials evoked strong cognitive dissonance, while self-easy trials evoked weak cognitive dissonance). In the computer trials, subjects chose the item that was selected by the computed algorithm, which was highlighted by a red square. In preference task II (Rating task 2), participants rated the same food items again.

Participants underwent a modified version of Brehm’s free-choice paradigms (Brehm, 1956) in the stimulation and sham sessions. The basic free-choice paradigm consisted of three main parts: (1) *preference task I*, (2) *choice task*, and (3) *preference task II*. Figure 1 illustrates the overall experimental design.

During *preference task I*, participants rated a set of 223 food items on an 8-point Likert scale (1 = “I do not like it at all” to 8 = “I like it a lot”). Each item was presented at the center of the screen for 3 s. The tDCS montage was set up, and active/sham tDCS was administered right after the end of *preference task I* and lasted for 20 min, during which, participants were instructed to sit comfortably on a chair.

During the *choice task*, each trial was formed by a pair of food items presented on the screen for 5 s. The trials were either *self*-choices, when participants make a decision themselves, or control *computer* choices, when participants had just to confirm the choice made by computer. In these choices, participants were not responsible for the choice and had not to experience dissonance. In *self-trials*, participants were instructed to select the preferred item by pressing the corresponding button on a computer keyboard. To enhance a participant’s motivation to select preferred items, participants were informed that they would receive one of the chosen items along with a show-up fee at the end of the experiment. The composition of each trial and the number of pairs in the choices were determined by the individual’s ratings of the items during the first *preference task I*. Participants were unaware that a computational algorithm used individual ratings to create the *self-trials*. Thus, we modulated choice difficulty by creating *self-difficult trials* that evoked high cognitive dissonance, as pairs were formed by highly preferred food items (rated between 6 and 8) and *self-easy trials*, which evoked low cognitive dissonance, since the pairs were formed by a highly preferred item and a poorly rated one (rated below 3). In the control *computer trials*, participants were instructed to press the button corresponding to the item which was randomly selected by the computer (highlighted by a red square). The *computer trials* were formed using the same criterion used to create *self-difficult trials*. All items were used only once during the *choice task*. At the beginning of each trial, participants were informed about the trial type (“your choice” or “computer choice”). Participants had 5 s to either choose an item or press the keyboard button corresponding to the computer’s choice. If there was no answer, a written message prompted participants to respond faster. Pairs in each choice condition were selected based on the participants’ ratings, thus the number of probes varied per person. On average, it reached 25 trials for difficult choices, 25 trials for easy choices, and 27 trials for computer choices.

During *preference task II*, participants rated the same set of food items. The only difference from *preference task I was an* additional message for items involved in the choice task. To be consistent with the previous studies (Izuma et al., 2010; Izuma and Murayama, 2013; Colosio et al., 2017) and to reduce the chance of participants forgetting their choice and to maximize the potential dissonance, these items were presented with a message informing the participant about which choice had been made (accepted or rejected item, e.g., “You rejected it”), either by the participant or the computer.

Finally, participants attended an additional control condition, namely a *post-ex (post-experimental) choice*. This task was introduced by Chen and Risen (2010) and was used in the work of Izuma et al. (2010) for control confounding preference changes. Chen and Risen noticed that re-evaluation of items could occur without the choice, followed by cognitive dissonance. In *post-ex* choice trials, as in *computer* trials in the *Choice* task, items were selected using the same criteria as in self-difficult trials. However, items, picked up for this *post-experimental* choice, were not assigned to any *self-choice* during the choice task. So, for these items, the order was “rate-rate-choose” instead of “rate-choose-rate” which eliminates confounding re-evaluations.

At the end of the experiment, we randomly selected one of the items that participants had selected during *self-difficult* trials or *post-ex* choice trials as an additional reward for the participants.

### 2.5 Statistical analysis

To evaluate how preferences were altered in decision-making, we compared the difference in items evaluation (ratings) between pre-choice *preference task I* and post-choice *preference task II* across different choice and stimulation types. The stimulation conditions comprised either active real stimulation (*cathodal tDCS* in Experiment 1 or *anodal tDCS* in Experiment 2) or *sham* (placebo stimulation). Types of choices (trials) included *difficult*, *easy*, and *post-experimental self-choices*, and *computer* choices. Choice types involved *rejection* or *selection* of items during the all types of choices. The mean of choice-induced preference changes served as the dependent variable and measured as the preference (rating) of the item in *preference task II* minus the preference (rating) of the same item in *preference task I*.

We aimed at modulating the re-evaluation process while making difficult self-choices under tDCS of the pMFC. We reasoned as follows: if the stimulation had an effect, then for items rejected in difficult self-choices, one would expect the decrease in preference changes under cathodal (inhibitory) tDCS (Experiment 1), and the increase in preference changes under anodal (excitatory) tDCS (Experiment 2) for the same kind of items, respectively, in comparison with sham stimulation. We also expected to observe the main effect of cognitive dissonance, i.e., stronger preference changes for items rejected during *self-difficult* choices (in general and separately in the target tDCS trials) as compared to selected ones and to items rejected in easy and computer choices (and no *post-ex* choices), which served as control conditions.

The main research hypothesis was to probe the modulatory effect of tDCS on preference changes (to reduce preference changes in Experiment 1 or to increase preference changes in Experiment 2). Taking into account the multiple-factor structure, the alteration of preference was only interesting when certain conditions were combined. The key point involved the comparison of mean choice-induced preference changes for items *rejected* in the *self-difficult* trials in the *tDCS* condition vs. those in the *sham* condition, using paired *t*-tests separately in each experiment. For the test of the general effect of cognitive dissonance, three separate paired t-tests were also performed. The tests compared mean preference changes in the tDCS condition for items *rejected* in the *self-difficult* trials vs.: (1) items *selected* in the *self-difficult trials*; (2) items rejected in the *self-easy trials*; (3) items rejected in the *computer trials*. All *t*-tests were performed with Bonferonni correction (alpha corrected = 0.05/4 = 0.0125). To assess whether changes in preference could reveal pre-existing preference rather than being associated with choice, we performed two-way 2 × 2 repeated measures ANOVA with two withing-subject factors: Choice (*rejected* or *selected*) and *Paradigm* (RCR, “rate-choice-rate” with *self-difficult* or *self-easy* choices), and RRC (“rate-rate-choice,” with *computer* and *post-ex choices*).

Next, for deeper investigation of the general effect of the tDCS on choice-induced preference changes, we performed the analysis of the mean preference changes for all the data obtained from both rejected and selected items using the linear mixed effects models (LME) (Bates et al., 2015a). In order to take into account individual differences, *Subject* was taken as a random factor, whereas *Stimulation* (cathodal tDCS vs. sham stimulation in Experiment 1 and anodal tDCS vs. sham stimulation in Experiment 2), *Trial type* (*self-difficult, self-easy, computer*) and Choice type (selected item vs. rejected item) were included as fixed factors. Post-experimental trials were not included here.

Data preprocessing and analysis was performed with R (R Core Team, 2022) in RStudio RStudio (RRID:SCR_000432) using R packages ‘data.table’ (Dowle and Srinivasan, 2019), ‘ez’ (Lawrence, 2016; RRID:SCR_020990), ‘lme4’ (Bates et al., 2015b; RRID:SCR_015654) ‘effsize’ (Torchiano, 2020), and ‘pwr’ (Champely, 2020). Visualizations were performed using the ‘ggplot2’ package (Wickham, 2016; RRID:SCR_014601). R-scripts for analysis and datasets are available on OSF.^1^

### 2.6 Linear mixed-effects model selection

The initial model design was chosen according to the principle of maximization random factor structure where all possible effects of random factors are considered using random intercepts and random slopes for the influence of all fixed factors (Barr et al., 2013). Estimation of maximal models, however, may not converge (Bates et al., 2015a). Taking into account the increased probability of getting type I error for random-intercepts-only models in within-subjects experimental design (Barr et al., 2013), the highest priority was given for models with both a random intercept and a random slope for at least one parameter. Further decisions about including random intercepts and random slopes for different fixed factors and goodness of fit of the model were made according to the model selection conditional Akaike Information Criterion (cAIC). cAIC provides special correction of estimation uncertainty of the random effects variance parameters based on a numerical approximation (Säfken et al., 2018). For coefficient estimates, the restricted maximum likelihood method (REML) was used instead of the maximum likelihood (MLE), which provides better computation in case of unbalanced design and unknown variance of random factors. It allows compare models with the same fixed factor and different random factors.

In both Experiment 1 and Experiment 2 cAIC showed the lowest (the best) value for the following model with correlated random intercept and slope, which has the structure Preference changes ~ Stimulation × Trial type × Choice type + (Stimulation|Subject). In a simplified form this model has formula:


$$\begin{array}{l} {\mathrm{PC}}_{si}=\beta_{0}+S_{0s}+\left(\beta_{1}+S_{1s}\right)\;{\mathrm{Stimulation}}_{i}\\ +\;\beta_{2}\;{\mathrm{Type}}_{i}+\beta_{3}\;{\mathrm{Choice}}_{i}+\beta_{4}\;{\mathrm{Stimulation}}_{i}\\ \times \;{\mathrm{Type}}_{i}+\beta_{5}\;{\mathrm{Stimulation}}_{i}\times {\mathrm{Choice}}_{i}\\ +\;\beta_{6}\;{\mathrm{Type}}_{i}\times {\mathrm{Choice}}_{i}+\beta_{7}\;{\mathrm{Stimulation}}_{i}\\ \times \;{\mathrm{Type}}_{i}\times {\mathrm{Choice}}_{i}+\varepsilon_{si;}\\ \varepsilon_{si}∼N\left(0;\sigma^{2}\right) \end{array}$$

where *β*_0_ − *β*_3_ – coefficients for intercept and slopes for fixed factors, *β*_4_ − *β*_7_ – coefficients for slopes for fixed factors interaction, S_0s_ and S_1s_ – coefficients for intercept and slope for random factor *Subject*.

Additional information regarding model selection is provided in Supplementary Material.

## 3 Results

The comparison of preferences for food items in A and B sets in pre-study proved that the sets had similar mean ratings (Set A = 4.70 ± 0.87; Set B = 4.69 ± 0.88). The independent *t*-test showed no significant difference between preferences for food items in sets A and B: *t*_(222)_ = 0.06, *p* = 0.94. The Shapiro–Wilk test for normality ensured that set A (*W* = 0.991, *p* = 0.215) and set B (*W* = 0.990, *p* = 0.121) were sampled from normal distribution.

### 3.1 Experiment 1. Effect of cathodal tDCS of the pMFC on choice-induced preference changes

**Figure 2:**
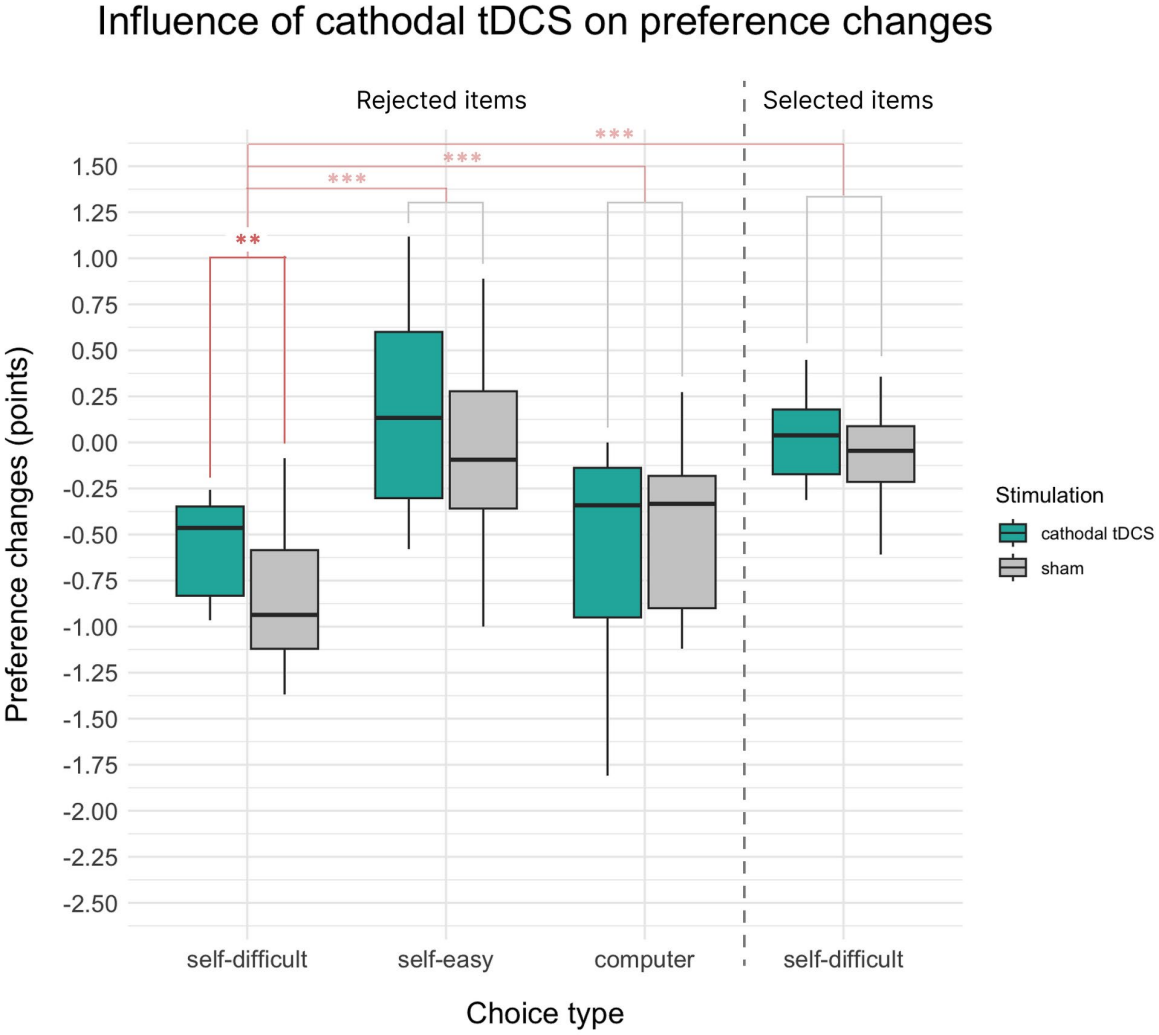
Mean choice-induced preference changes in Experiment 1 after cathodal tDCS or sham, indexed in points on an 8-point Likert scale. Target alteration of preference for items rejected in self-difficult choices under cathodal tDCS was smaller than after the sham condition (the median being closer to zero). The control comparisons of preference changes for items rejected in self-difficult choices were also significantly different from items selected in self-difficult choices and from items rejected in self-easy and computer choices. Significance level is indicated: *p* < 0.001 as ***, *p* < 0.01 as **, and *p* < 0.05 as *.

Paired *t*-test demonstrated that mean changes in preference for items rejected in self-difficult choices under cathodal tDCS were smaller than after sham condition (*t*(16) = −3.29, *p* = 0.002, Cohen’s *d* = 0.28, Hedges’s *g* = 0.27, one-sided). Figure 2 illustrates the result (the first two bars on the barplot), which confirmed our hypothesis: in self-difficult trials, cathodal tDCS significantly reduces choice-induced preference changes for rejected items compared to the placebo condition.

Preferences for *self-difficult* trials for the *rejected* items were significantly devalued comparatively to the *selected* ones (*t*(33) = −7.85, *p* < 0.001, Cohen’s *d* = 1.08, one-sided), which supports the general effect of cognitive dissonance. We also observed the significant difference between choice-induced preference changes for *rejected* items in target *self-difficult* trials and control *self-easy* trials (*t*(33) = −7.65.83, *p* < 0.001, Cohen’s *d* = 1.41, one-sided) and for *rejected* items in target *self-difficult* and control *computer* trials (*t*(33) = −3.33, *p* = 0.001, Cohen’s *d* = 0.57, one-sided). All these comparisons are shown in Figure 2. Two-way repeated measures 2 × 2 ANOVA Choice × Paradigm showed significant influence on preference changes for both factors Choice (*F*(1, 16) = 10.14, *p* = 0.006, *η*^2^*p* = 0.05) and Paradigm (*F*(1, 16) = 13.37, *p* = 0.002, *η*^2^*p* = 0.07), whereas interaction of Choice × Paradigm was insignificant (*p* = 0.22).

LME analysis (marginal *R*^2^_m_ = 0.25, conditional *R*^2^_c_ = 0.55) revealed significant contribution to preference changes on the subjects level of *Trial type* (*F* (2, 160) = 26.51, *p* < 0.001, *η*^2^_p_ = 0.25), Choice type (*F* (1, 160) = 8.58, *p* = 0.004, *η*^2^_p_ = 0.05), and their interaction of Trial type × Choice type (*F* (2, 160) = 22.57, *p* < 0.001, *η*^2^_p_ = 0.22). Other factors and interactions were not significant, including the target *Stimulation* factor (*p* = 0.23) and interaction of Stimulation type × Trial type × Choice type (*p* = 0.66). Coefficients estimates are provided in Table 1. ANOVA output on the LME model is shown in the Table 2. Descriptive statistics for mean choice-induced preference changes for items rejected in self-difficult choices under cathodal tDCS and sham are provided in the Supplementary Table S1.

**Table 1:** Experiment 1. Coefficient estimates of LME model with fixed factors stimulation, trial type, choice type, and random factor subject with correlated random intercept and random slope for stimulation.

Predictors	Estimates	CI	*p*
(Intercept)	−0.51	−0.81–−0.21	**0.001**
Stimulation_cathodal	−0.04	−0.37–0.28	0.812
Type_self-difficult	−0.58	−0.88–−0.27	**<0.001**
Type_self-easy	0.49	0.18–0.79	**0.005**
Choice_selected	−0.09	−0.39–0.22	0.607
Stimulation_cathodal × Type_self-difficult	0.29	−0.14–0.72	0.229
Stimulation_cathodal × Type_self-easy	0.26	−0.17–0.69	0.288
Stimulation_cathodal × Choice_selected	0.05	−0.38–0.48	0.844
Type_self-difficult × Choice_selected	1.07	0.64–1.50	**<0.001**
Type_self-easy × Choice_selected	−0.00	−0.43–0.43	0.997
Stimulation_Cathodal × Type_self-difficult × Choice_selected	−0.29	−0.90–0.32	0.394
Stimulation_Cathodal × Type_self-easy × Choice_selected	−0.24	−0.85–0.37	0.479

**Table 2:** Experiment 1. Results of ANOVA on LME model for choice-induced preference changes.

Fixed factor	Sum Sq	Mean Sq	Num Df	Den Df	*F*	*p*	*η*^2^*_p_*
Stimulation	0.1	0.1	1	16	0.47	0.5	
Trial type	15	5	3	224	24.34	<0.001***	0.25
Choice type	3.65	3.65	1	224	14.7	<0.001***	0.07
Stimulation × Trial type	0.34	0.11	3	224	0.55	0.65	
Stimulation × Choice type	0.21	0.21	1	224	1	0.31	
Trial type × Choice type	11.32	3.77	3	224	18.3	<0.001***	0.2
Stimulation × Trial type × Choice type	0.22	0.07	3	224	0.36	0.78	

LME model included fixed factors stimulation (cathodal tDCS vs. sham) × Trial type (difficult vs. easy vs. computer) and the choice type (rejected vs. selected) and random factor subject with correlated random intercept and random slope for stimulation. Significance level is indicated: *p* < 0.001 as ***, *p* < 0.01 as **, and *p* < 0.05 as *.

### 3.2 Experiment 2: effect of anodal tDCS of the pMFC on choice-induced preference changes

**Figure 3:**
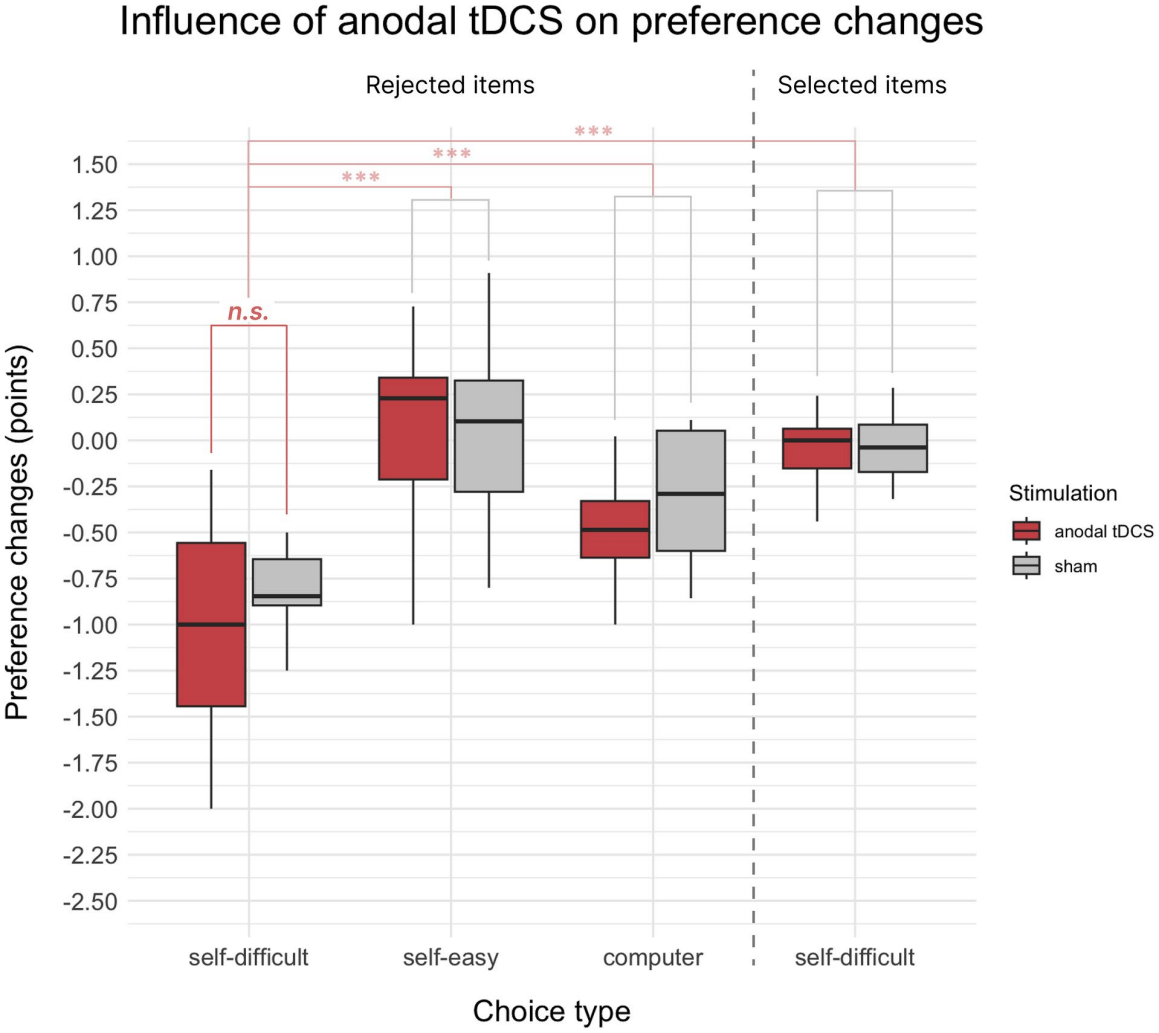
Mean choice-induced preference changes in Experiment 2 after anodal tDCS or sham, indexed in points on an 8-point Likert scale. Target alteration of preference for items rejected in *self-difficult* choices under anodal tDCS did not show a significant difference from the sham condition. The control comparisons of preference changes for items rejected in self-difficult choices were significantly different from items selected in self-difficult choices and from items rejected in self-easy and computer choices. Significance level is indicated: *p* < 0.001 as ***, *p* < 0.01 as **, and *p* < 0.05 as *.

Unlike Experiment 1, the paired *t*-test comparing preference changes for rejected in self-difficult choices items under anodal tDCS and sham stimulation did not reveal significant difference (*p* = 0.15). This result is illustrated by the left side of Figure 3 (the first two bars on the barplot).

As expected for having general cognitive dissonance effect, preference changes in *self-difficult* trials were significantly down for *rejected* items than for *selected* ones (*t*(37) = −11.73, *p* < 0.001, Cohen’s *d* = 2.08, one-sided). Also preferences changes for *rejected* items in *self-difficult* trials were significantly stronger than in *self-easy* (*t*(37) = −11.62, *p* < 0.001, Cohen’s *d* = 2.05) and *computer* trials (*t*(37) = −8.13, *p* < 0.001, Cohen’s *d* = 1.23). These comparisons are summarized and shown in Figure 3. Two-way repeated measures 2 × 2 ANOVA Choice × Paradigm showed significant influence on preference changes for both factors Choice (*F*(1, 18) = 30.24, *p* < 0.001, *η*^2^*p* = 0.34) and Paradigm (*F*(1, 18) = 6.15, *p* = 0.023, *η*^2^*p* = 0.03), whereas interaction of Choice × Paradigm was not significant (*p* = 0.67).

LME analysis (marginal *R*^2^_m_ = 0.4, conditional *R*^2^_c_ = 0.55) revealed significant contribution to preference changes on the subjects level of factors *Trial type* (*F* (2, 180) = 35.28, *p* < 0.001, *η*^2^*_p_* = 0.28), *Choice type* (*F* (1, 180) = 32.23, *p* < 0.001, *η*^2^*_p_* = 0.15), and their interaction Trial type × Choice type (*F* (2, 180) = 47.33, *p* < 0.001, *η*^2^*_p_* = 0.34). Other factors and interactions were also not significant, including the factor *Stimulation* (*p* = 0.1) and interaction of Stimulation type × Trial type × Choice type (*p* = 0.5). Table 2 provides the results of the LME analysis. Coefficients estimates are provided in Table 3. ANOVA output on the LME model is shown in the Table 4. Descriptive statistics for mean choice-induced preference changes for items rejected in self-difficult choices under cathodal tDCS and sham are provided in the Supplementary Table S2.

**Table 3:** Experiment 2. Coefficient estimates of LME model with fixed factors stimulation, trial type, choice type, and random factor subject with correlated random intercept and random slope for stimulation.

Predictors	Estimates	CI	*p*
(Intercept)	−0.30	−0.47–−0.14	**<0.001**
Stimulation_anodal	−0.23	−0.46–0.01	0.055
Type_self-difficult	−0.60	−0.80–−0.40	**<0.001**
Type_self-easy	0.34	0.14–0.54	**0.003**
Choice_selected	0.02	−0.18–0.22	0.865
Stimulation_anodal × Type_self-difficult	0.09	−0.19–0.38	0.571
Stimulation_anodal × Type_self-easy	0.20	−0.09–0.48	0.214
Stimulation_anodal × Choice_selected	0.11	−0.17–0.40	0.479
Type_self-difficult × Choice_selected	0.83	0.55–1.12	**<0.001**
Type_self-easy × Choice_Selected	−0.13	−0.41–0.16	0.432
Stimulation_anodal × Type_self-difficult × Choice_selected	−0.05	−0.46–0.35	0.817
Stimulation_anodal × Type_self-easy × Choice_selected	−0.24	−0.64–0.16	0.287

**Table 4:** Experiment 2. Results of ANOVA on LME model for choice-induced preference changes.

Fixed factor	Sum Sq	Mean Sq	Num Df	Den Df	*F*	*p*	*η*^2^*_p_*
Stimulation	0.35	0.35	1	18	3.47	0.08	
Trial type	8.83	2.94	3	252	29.34	<0.001***	0.25
Choice type	10.47	10.47	1	252	104.37	<0.001***	0.07
Stimulation type × Trial type	0.08	0.02	3	252	0.25	0.86	
Stimulation type × Choice type	0.02	0.02	1	252	0.17	0.68	
Trial type × Choice type	14.21	4.7	3	252	47.24	<0.001***	0.2
Stimulation type × Trial type × Choice type	0.16	0.05	3	252	0.55	0.65	

Here and below, LME model included fixed factors stimulation (cathodal tDCS vs. sham) × Trial type (difficult vs. easy vs. computer) and the Choice type (rejected vs. selected) and random factor Subject with correlated random intercept and random slope for Stimulation. Significance level is indicated: *p* < 0.001 as ***, *p* < 0.01 as**, and *p* < 0.05 as *.

The main results of two experiments and comparison of effect of cathodal and anodal tDCS in both experiments with its sham groups are summarized and illustrated in Supplementary Figure S1. Interpretation of comparison these results can be complicated due to individual differences between participants of studies: independent *t*-test showed no statistically significant difference in choice-induced preference changes in the *self-difficult* trials between anodal and cathodal stimulation in Experiment 1 and Experiment 2.

## 4 Discussion

In the current study, we used cathodal and anodal tDCS of the pMFC right before the *choice task* of the “free choice paradigm” to investigate the neural mechanism of the cognitive dissonance and subsequent choice-induced preference changes.

Regardless of the stimulation, we replicated a general behavioral effect of cognitive dissonance in both experiments: the preferences for items rejected in *self-difficult* (conflictual) choices significantly decreased after making the choice, compared to *self-easy* (non-conflictual) and computer (self-irresponsible) choices. This effect was observed regardless of the type of non-invasive tDCS: preference re-evaluation was detected in both Experiment 1 with inhibitory (cathodal) tDCS and Experiment 2 with excitatory (anodal) tDCS.

In Experiment 1, we observed that cathodal (inhibitory) tDCS of the pMFC particularly diminished choice-induced preference changes on declined options in difficult choices compared to the sham stimulation. This result supported the causal role of the pMFC in preference changes while making a difficult choice: suppressing the activity of the pMFC by cathodal tDCS prior to the choice reduced the reevaluation of the preference for rejected options. However, this result was demonstrated only in direct comparison using t-test, and effect size of the stimulation was comparatively small (Cohen’s *d* = 0.28). Further investigation of the general influence of cathodal tDCS on preference re-evaluation using linear mixed effects models (LME) did not show the significant effect of the tDCS.

In Experiment 2, we found no significant effect of anodal tDCS of the pMFC on the preference changes: neither in a focused analysis of the rejected items in *self-difficult* trials in comparison with sham condition nor analyzing data using linear mixed effect model.

One of the main possible reasons for not finding the strong effect of stimulation in two experiments as we expected is high probability of getting a false negative result. Having a limited knowledge of the neuromodulatory effects of tDCS on the activity of the pMFC in the cognitive dissonance studies poses difficulties to a prior calculation of the appropriate sample size in order to obtain reliable result. The posterior calculation of the statistical power, based on the observed effect size in Experiment 1, did not reach 80%, which makes a false negative outcome highly likely. Descriptive statistics (Supplementary Tables S1, S2) for choice-induced preference changes under stimulation and without it showed substantial heterogeneity and variability. We invited participants without neurological and psychiatric diseases and the use of any medication asked them not to drink coffee and alcohol on the day of the experiment and excluded those who experienced extreme fatigue and discomfort during the experiment. More attention should undoubtedly be paid to controlling the participants’ states in tDCS-experiments, due to the severe variability in the stimulation effect. These results can be used in subsequent tDCS-studies for prior calculation of the required sample size based on the statistical power and enhance the experimental design.

Another explanation of the current outcome in Experiment 2 is asymmetry in inhibitory-excitatory effects of non-invasive tDCS. This is supported by the results of a number of previous studies that have demonstrated heterogeneity of anodal and cathodal stimulation (Fregni et al., 2005; Karim et al., 2010; Mengarelli et al., 2015; for a meta-analysis see Jacobson et al., 2012). Some studies have specifically reported that there was no significant behavioral modulatory effect of anodal tDCS (Karim et al., 2010; Fagerlund et al., 2015; Conley et al., 2016). Further tDCS studies of conflict monitoring and resolution are needed to reconcile the asymmetry in stimulation effects and, in particular, to differentiate between the influence of anodal stimulation. Subsequent tDCS studies of choice-induced preference changes should pay specific attention to searching for the optimal target of brain stimulation. For example, evidence suggests that the more anterior subregions of the pMFC (FPz site) did not result in any modulatory effect on the ERN (Bellaïche et al., 2013). Systematic calculating of the electric field across many studies or the use of the high-definition tDCS could also be beneficial in reconciling the tDCS results across studies.

One further debatable point is the potential compensation of the effect of tDCS of the pMFC by the activity of unaffected brain areas, such as the dorsolateral prefrontal cortex (DLPFC). Recent investigations provide further evidence that a whole brain network is involved in the process of preference changes (Colosio et al., 2018; Voigt, 2022). For example, neuroimaging studies indicated an important role for the DLPFC in cognitive dissonance (Harmon-Jones and Harmon-Jones, 2008; Harmon-Jones et al., 2011; Mengarelli et al., 2015). Mengarelli et al. (2015) down-regulated the DLPFC by a 15 min, 1 mA cathodal tDCS. Offline stimulation of the left DLPFC significantly reduced the post-decision preference changes, and hence suggested that the left DLPFC plays an important role in the behavioral effects of cognitive dissonance. The role of the DLPFC in choice-induced preference changes is still under discussion, but it is thought to contribute to more general cognitive control mechanisms, regardless of whether conflicts is present (Harmon-Jones et al., 2011; Izuma et al., 2015). Interestingly, Ridderinkhof et al. (2004) proposed the existence of a functional pMFC-DLPFC network which supervises performance monitoring and executions. Further studies should focus on the development of possible controls for electromagnetic stimulation which can elucidate the interaction between the pMFC and DLPFC in choice-induced preference changes.

Although many neuroimaging studies demonstrated that the pMFC plays a central role in conflict monitoring, cognitive control and conflict resolution, little is certain about the chronometry of neuronal mechanisms of choice-induced preference changes. One of the first studies to show fMRI signatures of cognitive dissonance at the post-decisional stages in a “free choice paradigm” demonstrated that more conflicted decisions were associated with the larger pMFC activity during *preference task II*, compared to less conflicted decisions (Izuma et al., 2010). Of note, the majority of previous literature studying cognitive dissonance and choice-induced preference changes in the ‘free choice paradigm,” focused on the neural activity after decision during *preference task II* (Izuma and Murayama, 2013). For example, TMS of the posterior medial frontal cortex (pMFC) decreased preference changes only if applied at the later stages of the paradigm – right before *preference task II* (Izuma et al., 2015). However, relation between the pMFC and post-decisional preference changes is not always supported by experimental finding. For example, Kitayama et al. (2013) also showed elevated activity of the pMFC during making difficult conflictual choices (compared to easy ones), but found no correlation between the activity of the pMFC and post-decisional attitude changes. The neuroimaging study of Jarcho et al. (2011) examined the decisional phase of the decision-based cognitive dissonance paradigm and observed increased activity of the pMFC regions during the decision but not after it. Voigt et al. (2019) demonstrated, using fMRI and eye tracking, that activity of the DLPFC and pMFC, as well as and fixation duration during the making of hard decisions, predicted the magnitude of subsequent preference changes. Our study supports this evidence. Importantly, the duration of the tDCS after-effect is still a matter of debate: some studies have reported that a 20 min, 1.5 mA stimulation could generate a modulatory effect for several hours (Nitsche and Paulus, 2001; Nitsche et al., 2003; Reinhart and Woodman, 2014). In that case, in our study, cathodal tDCS could inhibit cortical activity during both the *choice task* and *preference task II*. Thus, new protocols should be developed to differentiate neural activity of the DLPFC and pMFC in the mechanisms of choice-induced preference changes.

Another interesting question is about the metacognitive aspects of preference changes in decision making. Some studies showed that at least partially preference changes can be attributed not to the fact of making difficult choices or the necessity of rearranging preferences to resolve conflicts, but rather to the internal refinement of the choice based on the certainty of pre-choice value judgment and confidence about the options in the decision (Lee and Daunizeau, 2020; Clairis and Pessiglione, 2022). Lee and colleagues provided a computational model for the online metacognitive control of decisions (Lee and Daunizeau, 2021; Lee et al., 2023). The fMRI study showed that value-based decision making and metacognitive evaluation of the option can be separated even at the neuronal levels (Clairis and Pessiglione, 2022). Therefore, it is important in future studies of the preference changing in making difficult decisions to disentangle the effects of the choice when comparing the expected values of the options and the subjective metacognitive process regarding this choice.

Generally, we traced the neuromodulatory (inhibitory) effect of cathodal tDCS on choice-induced preference changes. This effect was consistent with the proposed role and temporal dynamics of the pMFC: inhibiting the pMFC through cathodal tDCS, a key region in conflict detection and behavioral adjustments, prior to the making of a difficult decision, decreases the preference changes. This effect, however, was rather small, manifested only in direct comparisons with placebo stimulation and showed an asymmetry to the anodal (excitatory) tDCS, which did not demonstrate an increase in preference changes.

## Data availability statement

The original contributions presented in the study are publicly available. This data can be found here: https://osf.io/abpqj.

## Ethics statement

The studies involving humans were approved by Institutional Review Board of the National Research University Higher School of Economics. The studies were conducted in accordance with the local legislation and institutional requirements. The participants provided their written informed consent to participate in this study.

## Author contributions

ER: design of the study, data collection for experiment 2, statistical data analysis for experiment 1 and experiment 2, writing all sections of the manuscript, and figures and tables creation. MC: conceptualization and design of the study, stimuli development, and data collection for experiment 1. AS and VK: supervising the study and revision the manuscript. All authors contributed to the article and approved the submitted version.

## Footnotes

^1^https://osf.io/abpqj
