# Supplementary material for: Neuromodulation of choice-induced preference changes: the tDCS study of cognitive dissonance
Source: Front Psychol. 2023 Dec 18;14:1104410. doi: 10.3389/fpsyg.2023.1104410 (PMC10760977; doi:10.3389/fpsyg.2023.1104410)
Supplement: Supplementary file 1 [file Data_Sheet_1.docx]

Supplementary Material

# Additional information to linear mixed-effects model selection

The initial model design was chosen according to the principle of maximization random factor structure where all possible effects of random factors are considered using random intercepts and random slopes for the influence of all fixed factors (Barr, Levy, Scheepers, & Tily, 2013). ﻿ In order to take into account individual differences, *Subject* was taken as a random factor, whereas *Stimulation* (cathodal tDCS vs. sham stimulation in Experiment 1 and anodal tDCS vs. sham stimulation in Experiment 2), *Trial type* (*self-difficult, self-easy, computer*) and C*hoice type* (selected item vs. rejected item) were included as fixed factors. In our case, models with maximization of random factors structure were a model with random intercepts and slopes for all interacting fixed factors regarding random factor Subject, with correlated and uncorrelated intercepts and slopes. It has structure:

LME model1: Difference ~ Stimulation * Type * Choice + (Stimulation * Type * Choice | Sub)

LME model2: Difference ~ Stimulation * Type * Choice + (Stimulation * Type * Choice || Sub)

LME model3: Difference ~ Stimulation * Type * Choice + (Stimulation + Type + Choice | Sub)

LME model4: Difference ~ Stimulation * Type * Choice + (Stimulation + Type + Choice || Sub)

As was noted, the estimation of maximal models, however, may not converge (Bates et al., 2015). In our case, LME model 1 and LME model 2 had a problem with a number of observations that were less than a number of randoms factor, and return an error in model fitting. LME model 3 and LME model 4 turned out to be singular. To eliminate possible confounds in tDCS application caused by physiological individual differences (Miranda et al., 2018; Saturnino et al., 2019), random intercept and / or slope were chosen for fixed factor Stimulation. Models were probed with both correlated and uncorrelated intercept and slope, they had structure:

LME model5: Difference ~ Stimulation * Type * Choice + (Stimulation | Sub)

LME model6: Difference ~ Stimulation * Type * Choice + (Stimulation | | Sub)

LME model7: Difference ~ Stimulation * Type * Choice + (1 | Sub)

The further decision about model structure and goodness of fit of the model was made according to the model selection conditional Akaike Information Criterion (cAIC). cAIC provides special correction of estimation uncertainty of the random effects variance parameters based on a numerical approximation (Säfken, Rügamer, Kneib, & Greven, 2018).

cAIC for LME model5 = 386.79

cAIC for LME model6 = 386.81

cAIC for LME model7 = 393.49

The lowest (best) value of cAIC was for LME model 5, so, it was chosen as the final one. In a simplified form it has formula:

PC*_si_* = β_0_ +S_0s_ + (β_1_ + S_1s_) Stimulation*_i_* + β_2_ Type*_i_* + β_3_ Choice*_i_* + β_4_ Stimulation*_i_* *×* Type*_i_* +

β_5_ Stimulation*_i_* *×* Choice*_i_* + β_6_ Type*_i_* *×* Choice*_i_* + β_7_ Stimulation*_i_×* Type*_i_×* Choice*_i_* + ε*_si,_*_,_

ε*_si_* ~ N(0; σ^2^)

where β_0_ - β_3_ – coefficients for intercept and slopes for fixed factors, S_0s_ and S_1s_ – coefficients for intercept and slope for random factor *Subject*.

# Supplementary Figures


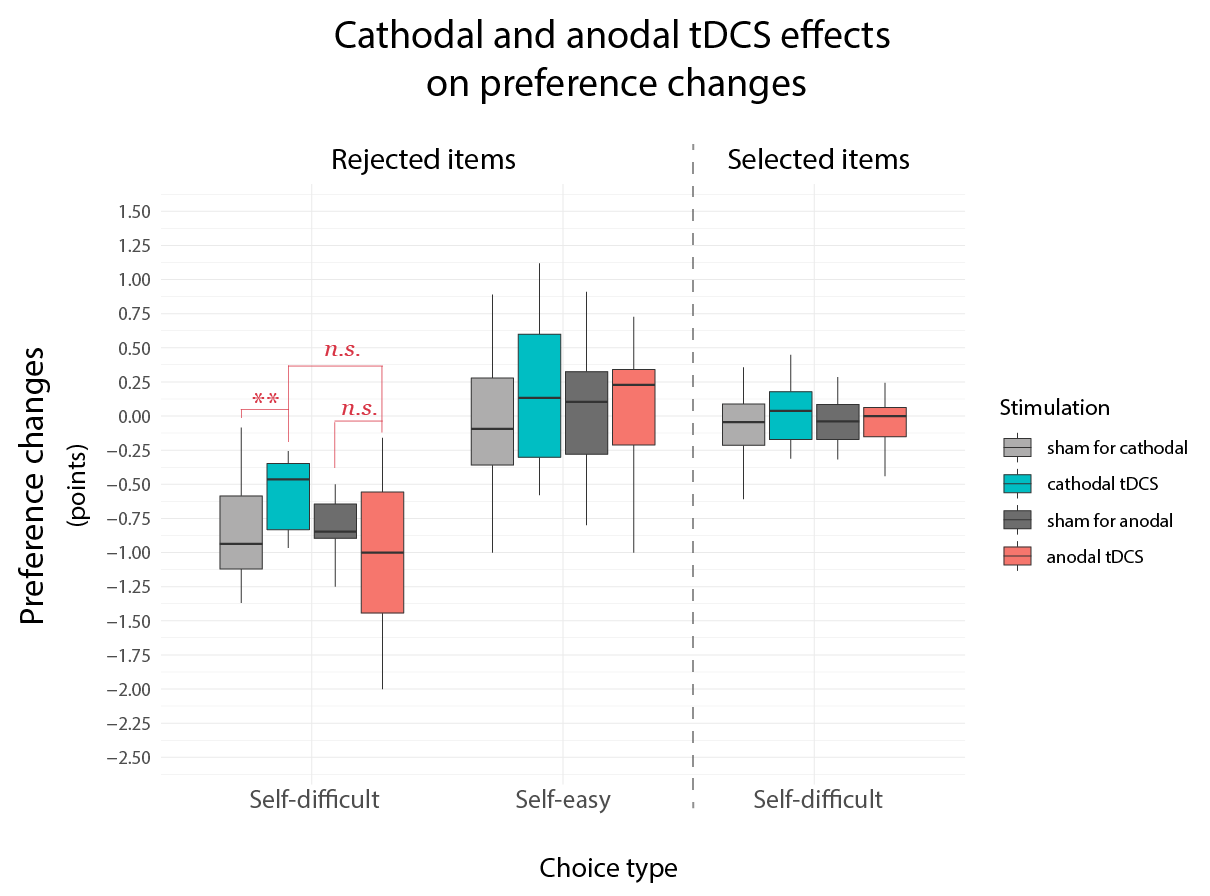


***Figure S1.*** Comparison of choice-induced preference changes for selected and rejected items in *self-difficult* and *self*-*easy* trials after cathodal or sham tDCS in Experiment 1 (left sides) and after anodal or sham tDCS in Experiment 2 (right sides)*.* Boxes representing stimulation conditions showed the opposite disposition of the medians for cathodal and anodal tDCS relatively to baseline sham, but independent t-test comparison did not show significant result.

# Supplementary Tables

**Table S1**

*Experiment 1 (cathodal tDCS). Descriptive statistics for mean choice-induced preference changes for items rejected in self-difficult choices under cathodal stimulation and sham condition*

| Stimulation | mean | median | sd | min | max |
| --- | --- | --- | --- | --- | --- |
| *cathodal tDCS* | -0.84 | -0.5 | 0.83 | -3.33 | -0.26 |
| *sham* | *-1.09* | *-0.94* | 0.89 | -4 | -0.09 |

**Table S2**

*Experiment 2 (anodal tDCS). Descriptive statistics for mean choice-induced preference changes for items rejected in self-difficult choices under anodal stimulation and sham condition*

| Stimulation | mean | median | sd | min | max |
| --- | --- | --- | --- | --- | --- |
| *anodal tDCS* | -1.04 | -1 | 0.58 | -2 | -0.16 |
| *sham* | *-0.9* | *-0.85* | 0.41 | -2.10 | -0.5 |
